# Characterizing the temporal Dynamics of Melatonin and Cortisol Changes in Response to Nocturnal Light Exposure

**DOI:** 10.1038/s41598-019-54806-7

**Published:** 2019-12-23

**Authors:** Shadab A. Rahman, Kenneth P. Wright, Steven W. Lockley, Charles A. Czeisler, Claude Gronfier

**Affiliations:** 1 Division of Sleep and Circadian Disorders, Departments of Medicine and Neurology, Brigham & Women’s Hospital, Boston, MA USA; 2 Division of Sleep Medicine, Harvard Medical School, Boston, MA USA; 3 Sleep and Chronobiology Laboratory, Department of Integrative Physiology, University of Colorado Boulder, Boulder, CO USA; 4 Lyon Neuroscience Research Center, Waking team, Inserm UMRS 1028, CNRS UMR 5292, Université Claude Bernard Lyon 1, Université de Lyon, F-69000 Lyon, France

**Keywords:** Circadian mechanisms, Neurophysiology

## Abstract

We studied the dynamics of melatonin suppression and changes in cortisol levels in humans in response to light exposure at night using high-frequency blood sampling. Twenty-one young healthy participants were randomized to receive either intermittent bright (~9,500 lux) light (IBL), continuous bright light (CBL) or continuous dim (~1 lux) light (VDL) for 6.5 h during the biological night (n = 7 *per* condition). Melatonin suppression occurred rapidly within the first 5 min and continued until the end of each IBL stimuli (t_1/2_ = ~13 min). Melatonin recovery occurred more slowly between IBL stimuli (half-maximal recovery rate of ~46 min). Mean melatonin suppression (~40%) and recovery (~50%) were similar across IBL stimuli. Suppression dynamics under CBL were also rapid (t_1/2_ = ~18 min), with no recovery until the light exposure ended. There was a significant linear increase of cortisol levels between the start and end of each IBL stimulus. Under CBL conditions cortisol showed trimodal changes with an initial linear activating phase, followed by an exponential inhibitory phase, and a final exponential recovery phase. These results show that light exposure at night affects circadian driven hormones differently and that outcomes are influenced by the duration and pattern of light exposure.

## Introduction

Melatonin secretion is acutely suppressed by light exposure at night^1,2^. While the intensity^3,4^, duration^5^ and spectral sensitivity^4,6–8^ of this response is well characterized in humans, the precise temporal dynamics of this response has not been studied in detail. Findings from studies of several animal models suggest that this response is rapid (*t*_1/2_ ~2–10 min)^9–14^, which is consistent with the rapid attenuation of enzymatic activity in the melatonin biosynthetic pathway^10,15^. The onset of melatonin suppression and the onset of melatonin recovery begin within ~5–15 min of the start and end of the light pulse, respectively. Recovery to baseline levels can be up to several hours in both humans and rats^11,16,17^. The suppression and recovery intervals in rats are accurate because high-frequency (every 15 min) microdialysis based sampling was used to measure melatonin in the extracellular space of the pineal gland^11^. In humans, however, the recovery-interval estimation of several hours may be less accurate since low-frequency sampling (hourly) was used after the light pulse ended^17^.

Unlike birds and reptiles^18–20^, the mammalian pineal gland is not directly light sensitive. Instead, a multisynaptic retinal-hypothalamic-pineal pathway carries photic signals from the eye to the suprachiasmatic nucleus (SCN), and to the pineal gland via the paraventricular nucleus of the hypothalamus, intermediolateral nucleus of the spinal cord and the superior cervical ganglion^21^. SCN lesion or transection of the multisynaptic pathway abolishes light-induced suppression of melatonin^22,23^. Another multisynaptic pathway has been identified leading from the SCN to the adrenal cortex^24^. Similar to melatonin, the circadian rhythm of glucocorticoids is also regulated by the SCN^25,26^. Light exposure in mice increases corticosterone levels via this sympathetic pathway without activating the hypothalamic-pituitary-adrenal axis^27^. The effects of light exposure on cortisol levels in humans are less clear, however. When humans were kept on a 3-h day, with 1 h of sleep in the dark and 2 h of wakefulness in the light in each 3-h interval, mean cortisol secretion was highest during the first hour of waking following the sleep interval^28^. Although this elevation in cortisol may have been the removal of sleep-induced suppression of cortisol^29^; nonetheless, the sleep to wake transition also corresponded with transitions from darkness to light, suggesting a stimulatory role of light exposure on human cortisol secretion. An increase in cortisol was reported during the first 15 min of the light exposure (2,000–4,500 lux) in the morning, although no change in cortisol in response to light exposure was observed during the evening^30^. Scheer *et al*.^31^, reported an increase in cortisol during the morning, within one hour after wake, but no change during an evening exposure using the same intensity (800 lux) and duration (1 hour) of light exposure. Bright light (5,500–10,000 lux) has been reported to induce a sustained decrease in cortisol during the descending^32,33^ and during the ascending phase of the cortisol rhythm^32^. Moreover, no change in cortisol levels have been reported in response to light exposure in other studies e.g., during a four-hour evening light exposure (5,000 lux)^34^, during a three-hour early night or late night exposure (5,000 lux)^35^, during a one-hour evening exposure (350 lux)^36^, or during a three-hour nighttime light exposure (600 lux)^37^. Therefore, the change in cortisol in response to light exposure appears to depend on the timing, the intensity, and possibly the duration of the light stimulus. Moreover, high-frequency sampling to assess the temporal dynamics of changes in cortisol secretion in response to light was not used in any of the studies noted above. Therefore, the aim of the current study was to investigate the precise temporal dynamics of changes in melatonin and cortisol levels induced by nocturnal bright light exposure.

## Methods

The general methodology has been reported in detail elsewhere^38^. We provide here the methods specific to the current analysis.

### Participants and pre-study conditions

Twenty-one healthy participants [24.6 ± 5.1 (SD) years old, 15 males, 6 females] were studied. The participants had no medical, psychiatric, or sleep disorders as determined by history, physical examination, electrocardiogram, blood and urine biochemical screening tests, and psychological screening questionnaires (Minnesota Multiphasic Personality Inventory and Beck Depression Inventory). A staff psychologist interviewed participants and those with a history of or a current psychiatric pathology were excluded. Participants reported that they were not taking any medication and were instructed to abstain from the use of alcohol, nicotine, recreational drugs, and foods or beverages containing caffeine for three weeks prior to the study. All participants were drug free at the time of study as verified by a comprehensive toxic analysis conducted upon admission to the laboratory. All experimental procedures were carried out in accordance with the principles of the Declaration of Helsinki, and the protocol was approved by the Human Research Committee at the Brigham and Women’s Hospital. Prior to beginning the protocol, all participants gave informed, written consent.

### Study protocol

Participants were required to maintain a regular 8 h:16 h sleep:wake schedule at home for at least 3 weeks prior to admission to the laboratory. In order to ensure compliance with this protocol, participants were required to call into a date/time-stamped answering machine just prior to going to bed and immediately upon awakening, and the times were compared to sleep-wake logs on the day of admission. In addition, wrist activity and light exposure were monitored for 1 week immediately prior to admission to the laboratory (Actiwatch-L, Mini Mitter, Sunriver, OR, USA) and were used to verify the stability of their sleep-wake cycle during that last week and throughout the entire impatient protocol.

**Figure 1:**
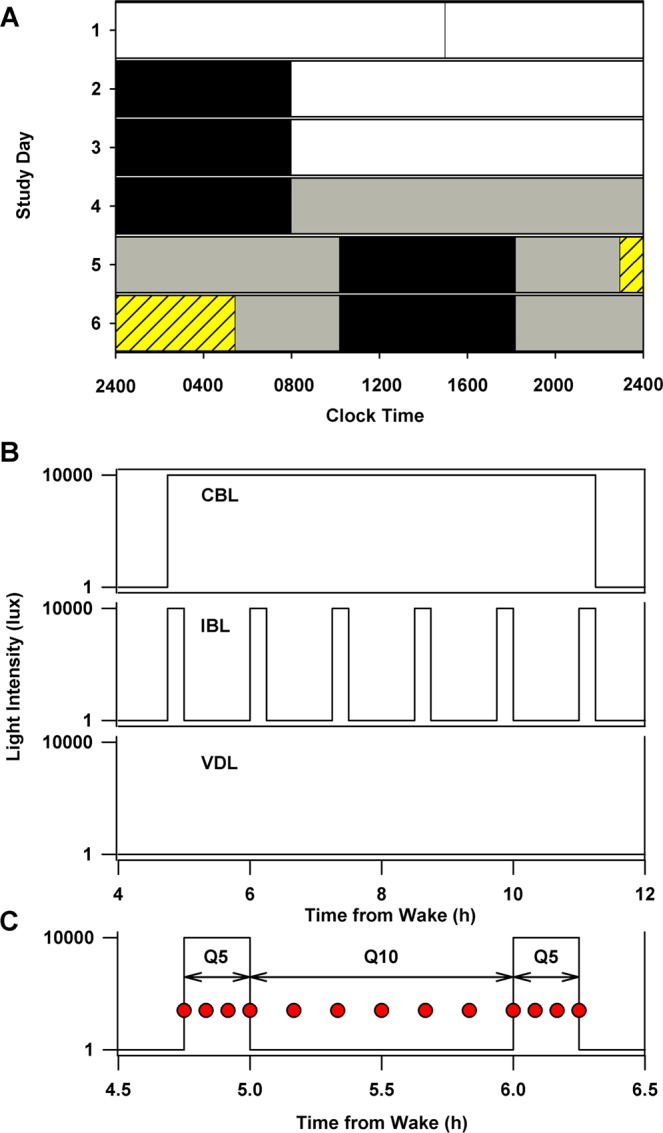
First 6 days of a 10-day experimental protocol and light exposure conditions. A representative study raster for an individual with 2400 h habitual bedtime and 0800 h habitual wake time (**A**). Scheduled sleep episodes (8 hours in darkness) are illustrated as black bars. During the baseline days (days 1–3), participants were exposed to ~90 lux during wakefulness (16 hours; white bars). For the remainder of the study, except during the light exposure session, participants were exposed to ~1.5 lux during wakefulness (grey bars). A ~ 26.2-h constant routine was scheduled on days 4–5. The light exposure session (yellow hashed bar) was scheduled on days 5–6, and consisted of 6.5 hours of exposure centered 5.8 hours before habitual wake time. Following the light exposure day, participants underwent a 64-h CR and were discharged after a ~22-h recovery sleep episode (days 7–10; not shown). Light exposure conditions (**B**): participants were exposed either to 6.5 hours of continuous bright light (CBL; ~9,500 lux), intermittent bright light [IBL; six 15-minute bright light (~9,500 lux) stimuli separated by 60 minutes of very dim light (<1 lux)] or very dim light (VDL; < 1 lux). High-frequency blood sampling used to assess melatonin and cortisol dynamics under the different light conditions, every 5 min (Q5) during the 15 min light exposure stimulus or every 10 min (Q10) during the 60 min recovery interval (**C**). The sampling frequency and patterns were the same under all three light exposure patterns.

Upon admission to the study on experimental day 1, participants were maintained in an environment free from external time cues, including clocks, radios, television, visitors, and sunlight. Participants were continuously monitored by staff members specifically trained to avoid communicating time of day or the nature of the experimental conditions to the participants. Participants were adapted to the laboratory with three baseline days (Days 1–3), during which time they continued to sleep and wake at their habitual times (Fig. 1A). In order to assess their endogenous circadian phase before the light stimulus and to center the light stimulus during the following scheduled episodes of wakefulness, participants underwent a 26.2-h initial constant routine (CR1; procedure described below) from day 4 to day 5. The duration of the constant routine was designed so that the center of the 6.5-h light exposure session was 5.8 h before habitual wake time. On day 5, participants were randomly assigned to one of the three light exposure conditions (described below).

During wake episodes, participants were free to move about the suite as desired, except that they were asked not to lie down, nap, or exercise. Participants’ compliance with the protocol was monitored by means of closed-circuit cameras and frequent interaction with technicians. The experimental suites were equipped with ceiling-mounted 4100 K fluorescent lamps [Phillips, Eindhoven, The Netherlands; additional details on light measurements and light spectrum reported in^39^]. A computer system automatically turned the lighting to the required pre-set intensity at the scheduled times. Maximum light intensities, as measured in the horizontal plane at a height of ~1.83 m using an IL1400 photometer (International Light, Newburyport, MA), were ~190 lux (~48 µW/cm^2^) during the waking hours of the three baseline days [~90 lux (~23 µW/cm^2^) measured in the vertical plane at a height of ~1.37 m]; < 8 lux [~1.5 lux ( < 0.4 µW/cm^2^ to ~0.1 µW/cm^2^) measured in the vertical plane at a height of ~1.37 m] during the constant routines and the day of light exposure; and depending on the experimental condition (described below), < 1 lux [~0.5 lux (0.1 µW/cm^2^) measured in the vertical plane at a height of ~1.37 m] or ~9,500 lux (~8 × 10^6^ µW/cm^2^ in the direction of gaze at a wall-mounted target) during the light exposure session. Sleep episodes were conducted in darkness.

### Constant routine procedure

A constant routine (CR) procedure^40^ was used in the current analysis to assess baseline levels of melatonin and cortisol under dim light conditions on the day prior to light exposure. The CR consisted of a regimen of enforced wakefulness in a semi-recumbent posture in constant dim illumination of ~1.5 lux. Participants were required to maintain a very low level of physical activity and were not permitted to change their posture throughout the constant routine. This posture was also maintained for urine samples and bowel movements. Nutritional intake was divided into hourly isocaloric snacks to maintain an equal caloric intake across the circadian cycle. Caloric requirements were calculated with use of the Wilmore nomogram^41^ to determine the basal metabolic rate, and adjusted upward by a 10% activity factor to account for increased energy expenditure during sleep deprivation of the constant routine^42^. A staff member monitored the participant to help maintain wakefulness and to ensure compliance with the posture and activity level requirements.

### Light exposure conditions

Participants were randomly assigned to one of the three light exposure conditions (Fig. 1B). The three conditions were: continuous bright light (BL) of ~9,500 lux (~2,900 µW/cm^2^), defined as 100% bright light; intermittent bright light (IBL) consisting of six 15-minute bright light stimuli of ~9,500 lux [corresponding to ~6700 melanopic lux^43^] separated by 60 minutes of very dim light [<1 lux (~0.1 µW/cm^2^)], defined as 23% bright light; and continuous very dim light (VDL) of <1 lux, defined as 0% bright light. In all three conditions, the 30 min prior to and the 30 min following the light exposure session were conducted in <1 lux. Seven participants were randomized to each condition. Participants were seated in a chair from 2 h before until 2 h after the end of the light exposure session. A technician was present at all times during the light exposure session to ensure compliance with the protocol. During the light exposure sessions, participants were instructed to fix their gaze on a target mounted on the wall of their suite for 5 of every 10 min and then allowed free gaze for 5 of every 10 min to ensure consistency of light exposure between participants. This alternating fixed and free gaze episodes continued during the entire duration of the light exposure sessions. Light intensity measurements were taken in the direction of gaze every 5 min to ensure that participants were exposed to the targeted light intensity for the appropriate duration. Clear polycarbonate lenses filtered 99.9% of the light in the UV range from the light source. In addition, all participants wore clear goggles (model #S379; luminous transmittance 90%, UV absorption >99%, Uvex Safety, Smithfield, RI, USA) during the exposure sessions to bright light for additional protection against UV exposure.

### Melatonin and cortisol assays

Blood samples were collected every 5 to 10 min during the light exposure session (Fig. 1C), through an indwelling intravenous catheter that was inserted into a forearm vein on day 2 of the study. A solution of heparinized saline (0.45% sodium chloride, 10 units of heparin/ml) was infused at a slow rate (20 ml/h, 200 IU heparin/h) between blood samples. We provided participants with ferrous gluconate (324 mg) pills to be taken at breakfast and dinner: (1) for a minimum of one week prior to participation in the inpatient research protocol; (2) during the inpatient portion of the protocol; and (3) for three weeks after completion of the protocol. Participants’ hemoglobin levels were tested every 1–2 day(s) to ensure appropriate levels (>11.0 for men, >10.3 for women). Blood samples were collected in ethylenediaminetetraacetate (EDTA)-K2 tubes, rapidly centrifuged at 4 °C, and the plasma was stored at or below −25 °C until assayed. Samples were assayed for melatonin using radioimmunoassay techniques (Diagnostech/Pharmasan, Osceola, WI, USA). The assay sensitivity was 2.5 pg/ml. The average intra-assay coefficients of variation (CVs) were 6.4% below 50 pmol/L, and 4.9% above. Plasma cortisol levels were determined by chemiluminescent assay (Beckman Coulter, Chaska, MN, USA); sensitivity, 0.4 mg/dL; intra- and inter-assay coefficients of variation, 6.4% and 7.9%, respectively.

### Data analysis

Data are presented as mean ± SD unless stated otherwise. Melatonin and cortisol time course data were analyzed using both between-condition [data compared between individuals exposed to light exposure (IBL and CBL) and dim light conditions] and within-participant by condition analysis [data compared between light exposure (IBL and CBL) and dim light conditions under CR 24 h earlier] to ensure analytic robustness. The within-participant by condition analysis accounts for intra-individual differences in circadian phase, which may have affected hormone levels. In contrast, the between-condition analysis does not account for intra-individual differences.

Time-series melatonin and cortisol data were analyzed using two-way repeated measures with random intercepts mixed model analysis of variance using the restricted maximum likelihood estimation method (REML); fixed effects were set as light-exposure condition and duration of exposure. Simple main effects analyses were used to determine the effect of one factor separately for each level of the other factor. If a significant main or interaction effect was observed, then pairwise comparisons followed using 2-sided t-tests^44^.

To estimate the effects of each light stimulus on melatonin and cortisol levels, the percentage change from the start to the end of each light stimulus were determined. Similarly, to estimate recovery in melatonin and cortisol levels during the dim-light interval between light stimuli, the percentage change from the end of a light stimulus to the start of the next light stimulus were determined. To adjust for changes in hormone levels with circadian phase, pre- and post-light stimulus values were normalized to melatonin levels by expressing them as a ratio of the levels under dim light conditions 24 h earlier during CR, for each individual. The individual dim-light adjusted percentage-change values were subjected to outlier detection using 2-sided Rosner’s Extreme Studentized Deviate test for multiple outliers with a *p*-threshold of 0.01^44^. Three out of 42 data points were detected as outliers in the recovery dataset (2 points in the first stimulus and 1 point in the last stimulus) and none in the suppression dataset. Outliers were removed from further analysis. The individual dim-light adjusted values were then averaged across individuals and analyzed with one-way (stimulus number) repeated measures generalized linear models.

Additionally, accounting for the pulsatile secretion dynamics of cortisol and melatonin, we explored the linear change (increasing or decreasing) in melatonin and cortisol levels in response to bright light by linear regression of three consecutive points (concentrations at t = 5, 10 and 15 min) corresponding to 5 min after lights onset to the end of the light stimulus in the IBL condition, across all 6 stimuli. A positive slope corresponded to an increasing change in the levels between the start and end of the light stimulus, whereas a negative slope corresponded to a decreasing change. The number of positive and negative slopes during each stimulus was compared statistically using binomial probability tests.

To assess melatonin dynamics during and between each light stimulus, data were first expressed as a percentage of the fitted peak under CR for each individual and then averaged across individuals, for each light stimulus separately. This time series data were fitted with exponential-decay regression models [f(x) = a * e^(−b^ * ^x)^] during the light stimulus and 4-parameter logistic regression models [f(x) = ((a − b)/(1 + (x/c)^d^)) + b] during recovery (dim light between stimuli).

To assess the dynamics in melatonin and cortisol during continuous light exposure, data were first expressed as a percentage of the fitted peak under CR for each individual and then averaged across individuals. Melatonin time course data were fitted with a 3-parameter exponential-decay regression model [f(x) = (a − b) * e^(−c^ * ^x)^ + b]. Cortisol time course data appeared to be triphasic and therefore a piece-wise fit was used with a linear model [f(x) = b + ax] for the first ~100 min, a 3-parameter exponential-decay model [f(x) = (a − b) * e^(−c^ * ^x)^ + b] for the next ~100 min, and a 3-parameter exponential growth to maximum regression model [f(x) = a + b * (1-e^(−c^ * ^x)^)] for remainder of the 6.5 hour light exposure. To compare the dynamics of decreasing melatonin levels following the start of continuous bright light exposure to the decrease in melatonin levels following the cessation of melatonin synthesis (*SynOff*)^45,46^, the t_1/2_ from the exponential decay phase under both conditions were compared using a F-test. The rate of decrease in melatonin levels following *SynOff* was calculated by applying a 3-parameter exponential-decay regression to the group mean melatonin profile derived under dim-light conditions during CR in the 24 h prior to the bright light exposure. *SynOff* was defined as previously described^45^ and could be calculated in 5 out of the 6 individuals who were exposed to continuous bright light.

## Results

### Melatonin and Cortisol Secretion Profiles

**Figure 2:**
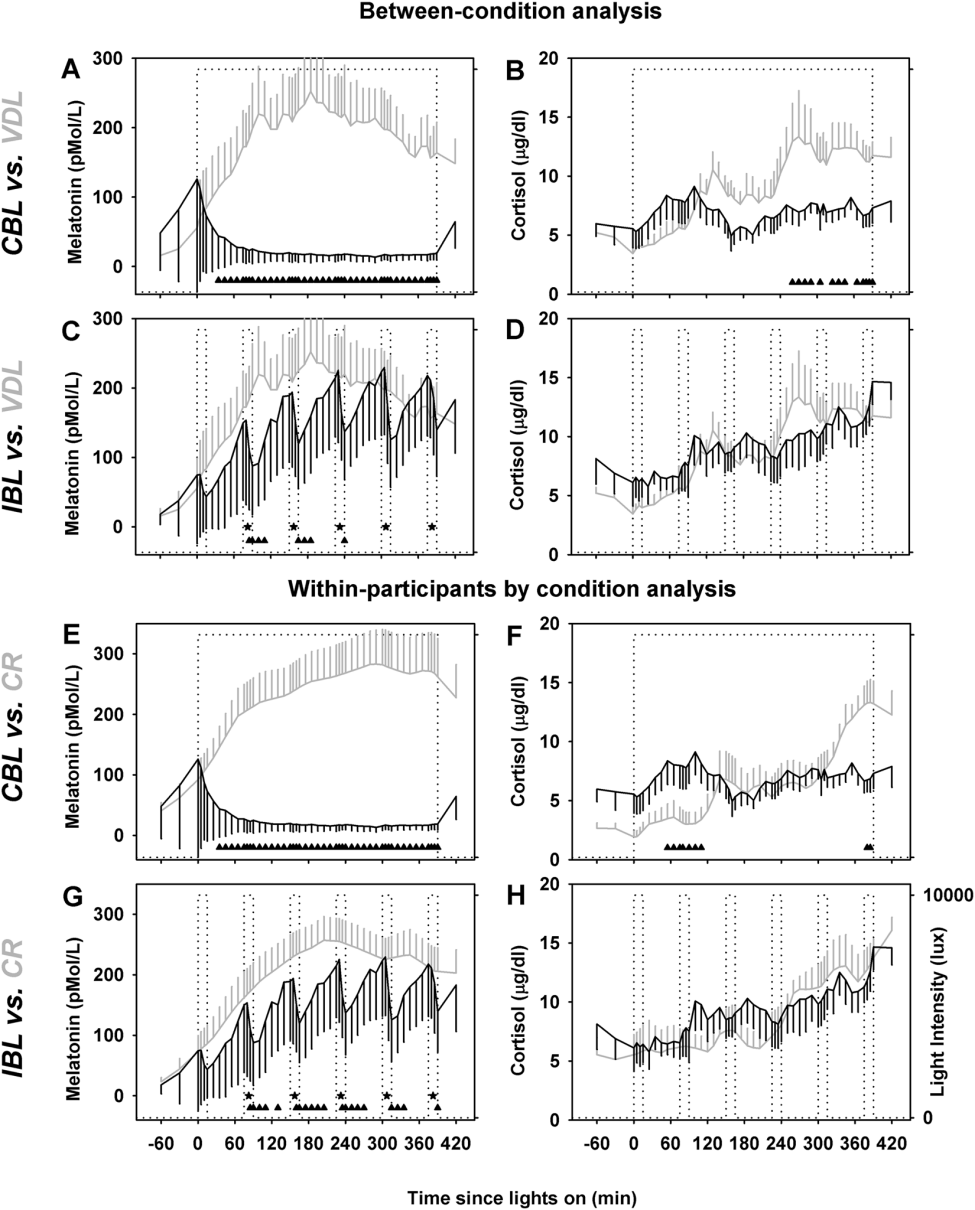
Circulating melatonin and cortisol profiles under different lighting conditions. Melatonin and cortisol profiles under very dim light (VDL) and under constant routine (CR) conditions are shown in gray. Profiles under continuous bright light (CBL) exposure for melatonin (**A**) and cortisol (**B**) and intermittent bright light (IBL) exposure for melatonin (**C**) and cortisol (**D**) using between-conditions analysis and continuous bright light (CBL) exposure for melatonin (**E**) and cortisol (**F**) and intermittent bright light (IBL) exposure for melatonin (**G**) and cortisol (**H**) using within-participant by condition analysis. Light exposure patterns are shown as dotted lines. Significant differences in melatonin and cortisol levels between VDL or CR and CBL (A and B) or IBL (C and D) at specific exposure times are shown as ▲. Data shown are group mean (±SEM). Significant differences between pre- and post-light exposure levels of melatonin and cortisol levels under IBL condition is shown as *.

Melatonin suppression under the IBL and CBL conditions has been partially reported previously^5,45,47^. Using a between-condition analysis [data compared between individuals exposed to light exposure (IBL and CBL) and dim light conditions], we found that the melatonin levels were significantly different between dim light and continuous bright light (CBL) or intermittent bright light conditions (*p* < 0.01). As expected, melatonin levels increased across time under dim light conditions, and were suppressed throughout the entire duration of the CBL exposure (Fig. 2A) and by IBL stimuli (Fig. 2C) as revealed by a significant interaction between exposure duration and condition (*p* < 0.001). Simple main effects analyses of this interaction by t-tests showed that, compared to dim light, melatonin levels were lower under CBL at all times starting ~30 min after the onset of the bright light exposure. Additionally, melatonin levels during three of the six IBL stimuli were significantly lower as compared to the dim light condition (Fig. 2C). Moreover, examining the levels of melatonin at the start and end of the light stimulus showed that melatonin levels were significantly decreased at the end of the stimulus compared to the start of the stimulus during the last five IBL stimuli (Fig. 2C).

Similar to melatonin, cortisol levels were significantly different between dim light and CBL exposure. Cortisol levels were significantly lower under CBL as compared to the dim light condition during the latter half of the light exposure, which corresponded to the rising phase of the cortisol profile under dim light (Fig. 2B). In contrast, cortisol levels were not significantly reduced during IBL stimuli (Fig. 2D). Neither comparing cortisol levels during each light-stimulus interval in IBL and dim-light conditions, nor comparing cortisol levels at the start and end of each light stimulus showed significant differences (Fig. 2D).

Within-participant by condition analysis [data compared between light exposure (IBL and CBL) and dim light conditions under CR 24 h earlier] showed results mostly similar to the between-conditions analysis. One difference was that the melatonin levels were significantly lower during the last five IBL stimuli compared to the levels under the dim light condition using the within-participants by condition analysis (Fig. 2G), whereas only three pulses were different using the between-condition analysis (Fig. 2C). For cortisol, the only difference between the two types of analyses was that as compared to cortisol levels under dim light conditions, there was a significant increase in cortisol levels under CBL condition beginning about 1 h after the onset of bright light exposure and continuing for ~40 min during approximately the first quarter of the light exposure corresponding to the quiescent phase of the cortisol rhythm (Fig. 2F).

### Stimulus-Wise Change in Melatonin and Cortisol under Intermittent Bright Light Exposure

**Figure 3:**
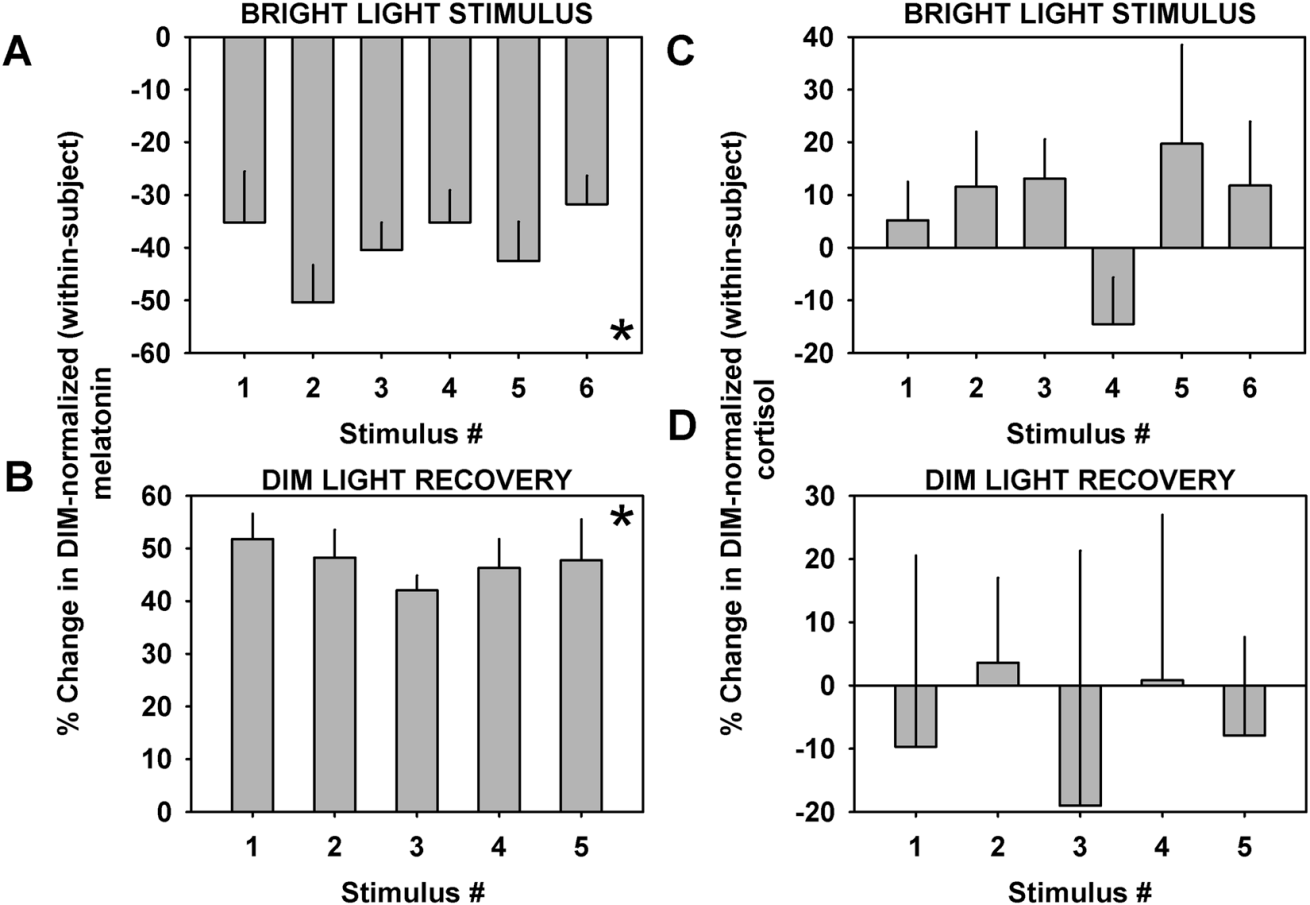
Change in melatonin and cortisol levels during the light stimulus and recovery interval for each individual bright light stimulus. Change in melatonin during the light stimulus (**A**) and recovery intervals (**B**) and in cortisol during the light stimulus (**C**) and recovery intervals (**D**) observed with each 15-min stimulus during the intermittent light exposure pattern. Data shown are group mean (±SEM). Melatonin levels were significantly suppressed and recovered between pre- and post-light and post- and pre-light stimulus, respectively *. Cortisol did not show significant changes associated with any of the six pulses.

Melatonin levels were on average suppressed by ~40% (Fig. 3A) during each intermittent light stimulus, and recovered by ~50% (~1.5 times increase) between the end of one stimulus and the start of another (Fig. 3B) in the IBL condition. Melatonin levels significantly changed from zero during all of the IBL stimuli (*p* < 0.001) and during recovery (*p* < 0.0001). There were no statistically significant differences in the level of suppression (*p* = 0.50) and recovery (*p* = 0.85) (one-way GLM) between each of the stimuli after adjusting for the physiological increase in melatonin levels associated with changing circadian phase. There was no significant change in average cortisol levels during each intermittent light stimulus (Fig. 3C), or during recovery (Fig. 3D).

Additionally, the inhibitory effects of light stimuli on melatonin was confirmed by that 95.3% (40 out of 42) of the intermittent bright light stimuli resulted in an acute linear decrease of melatonin levels during the light pulse (binomial probability test; *p* < 0.0001, significant association). In contrast, cortisol concentrations increased linearly during the light pulses for 71.4% of the bright light stimuli (30 out of 42; binomial probability test; *p* < 0.005, significant association). As a control, melatonin and cortisol increased or decreased linearly at random at the same times during the CR 24 h earlier under dim light (54.8% increases and 45.2% decreases for melatonin, p = 0.64; 47.6% increases and 52.4% decreases for cortisol, *p* = 0.88, no association).

### Melatonin Suppression and Recovery Dynamics to Intermittent Bright Light Exposure

**Figure 4:**
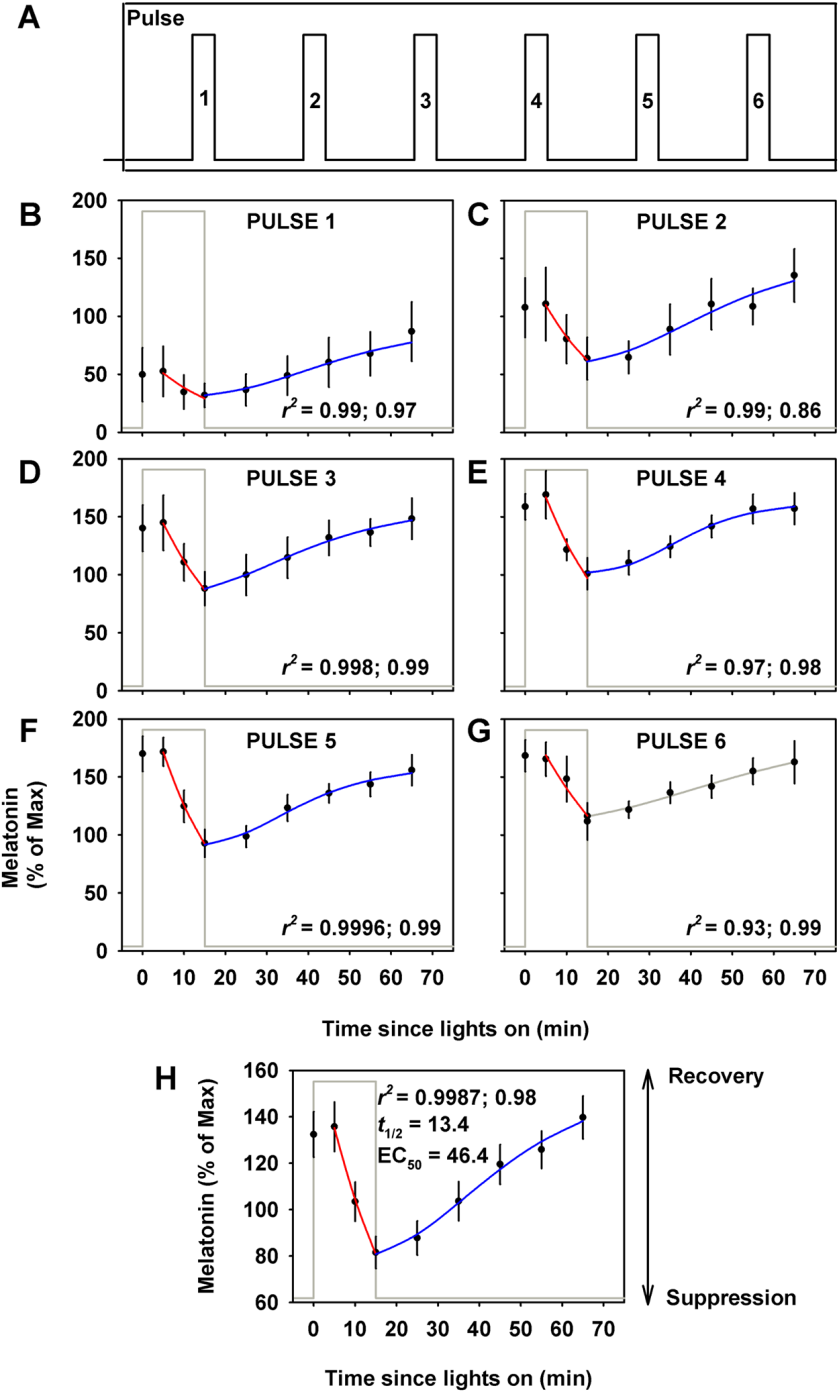
Melatonin suppression and recovery dynamics. Linear and non-linear regression models were applied to group mean (±SEM) melatonin levels under intermittent light exposure. Stimulus pattern under the intermittent condition is shown for reference (**A**). Suppression regression-line is shown in red and recovery regression-line is shown in blue for each individual stimulus (**B–G**) and the group mean profile across all six stimuli (**H**). The recovery time-course for the last stimulus was under dim light after the 6.5 h exposure interval ended (gray line) and is shown for illustrative purposes and was not included in the recovery time-course profile analysis in (H). Adjusted *r*^*2*^ values from the regression analyses are shown for the suppression followed by the recovery interval; *t*_*1/2*_: half-life of suppression; EC_50_: half-maximal recovery. Data shown are group mean (±SEM).

Group mean profiles were generated for each of the six stimuli individually (Fig. 4B–G) and across all six stimuli (Fig. 4H) using Q5 min sampling for melatonin during the 15-min light stimulus and Q10 min sampling during the 60-min recovery interval. Besides the first stimulus, all remaining five stimuli during the suppression interval were fit significantly using an exponential-decay regression model. The mean half-life (*t*_*1/*2_) across six stimuli was 13.5 ± 2.9 min. The *t*_*1/2*_ derived from the group mean profile across all six stimuli was 13.4 ± 1.3 min (Fig. 4H).

Besides the second recovery interval, all remaining five recovery intervals were fit significantly using the logistic regression model. The mean EC_50_ across all five recovery intervals was 46.4 ± 11.3 min. The EC_50_ calculated from the recovery phase of the group mean profile was 46.4 ± 28.2 min (Fig. 4H).

### Melatonin and Cortisol Responses to Continuous Bright Light Exposure

**Figure 5:**
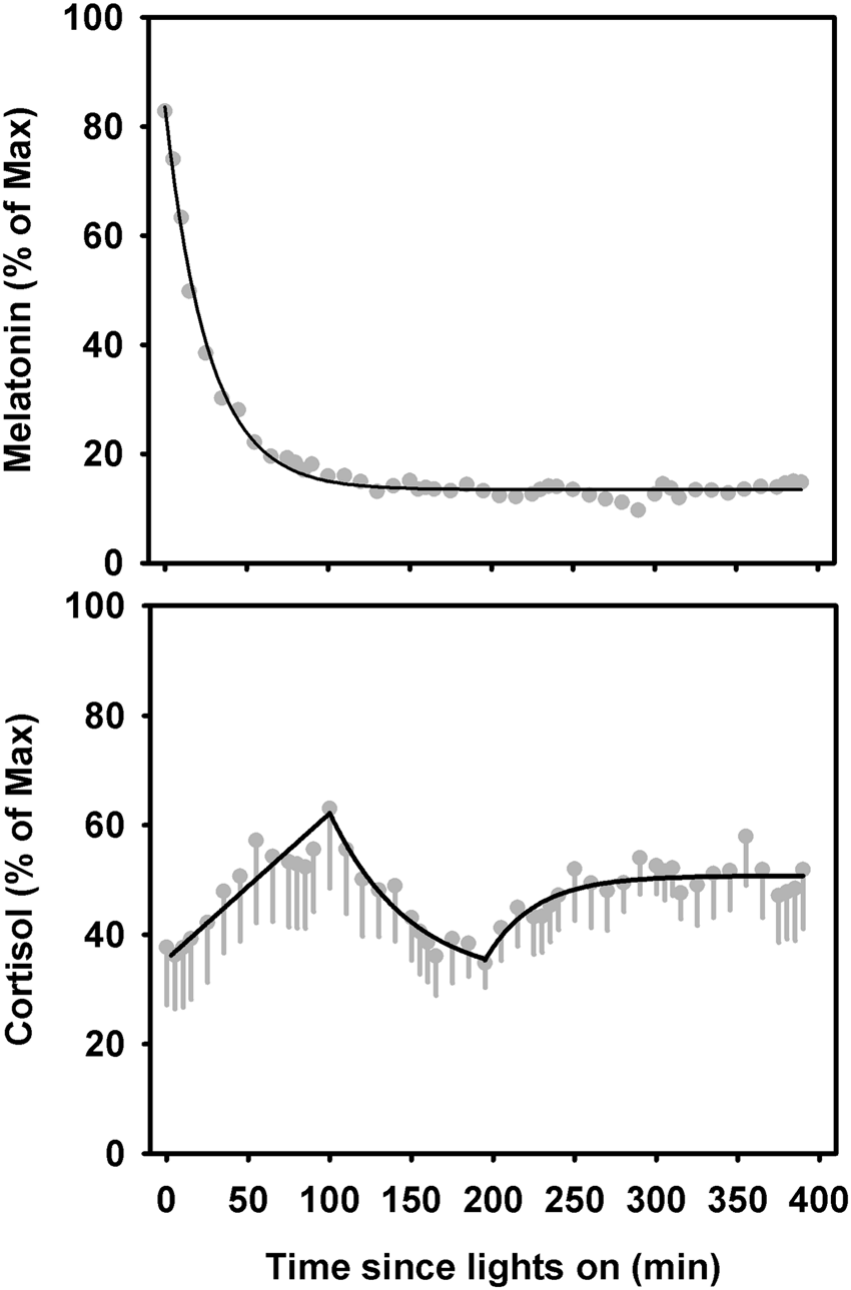
Change in melatonin and cortisol in response to continuous bright light exposure. Change in melatonin (**A**) and cortisol (**B**) as a function of duration of exposure under the CBL condition. Data shown are group mean (±SEM).

Under continuous bright light exposure there was a sustained suppression of melatonin across the entire light exposure duration (Fig. 5A). This response was best fit by an exponential decay regression model, applied to the group mean profile, which showed a half-life of 18.2 ± 1.4 min. This rate was significantly faster than the rate of melatonin decrease following estimated *SynOff* (56.2 ± 9.6 min, p < 0.0001). In contrast, cortisol levels under continuous bright light exposure showed trimodal dynamics. In the first ~100 min cortisol levels increased linearly (3.7% increase per minute of exposure) followed by an exponential decrease (*t*_*1/2*_ = 34.0 ± 26.3 min) over the next ~100 min and a final exponential recovery (*t*_*1/2*_ = 21.4 ± 14.5 min) phase for the remainder of the light stimulus (Fig. 5B).

## Discussion

In the current study we used high-frequency sampling under continuous and intermittent light exposure patterns to characterize the dynamics in melatonin and cortisol changes in response to nocturnal light exposure. Our results show that melatonin and cortisol secretion are differentially affected by nighttime light exposure. The change in melatonin levels is rapid, sustained and consistent across the continuous and intermittent light exposure patterns. The change in cortisol, however, is complex and appears to change based on the duration of light exposure. There is a linear increase in cortisol levels in the first ~15 minutes of light exposure, which is sustained for up to ~1.7 h of the continuous light exposure. Following that initial increase, cortisol levels decrease exponentially for an additional ~1.7 h, and finally stabilize during the final ~3 h of the continuous light exposure.

Since the changes in melatonin levels were sustained and consistent across light exposure patterns, we further assessed the dynamics of this response. Under the intermittent exposure pattern, melatonin levels reached half maximal suppression in ~13 min after the start of the 15-min light stimulus and recovered to half-maximal levels in ~29 min after the end of the light stimulus. Melatonin suppression and recovery was similar for all stimuli, after accounting for endogenous changes in the levels of melatonin caused by changes in circadian phase.

Under the continuous exposure pattern, melatonin levels reached half maximal suppression in ~18 min after the start of the 6.5-h light stimulus and did not recover until after the end of the light stimulus. Although the *t*_*1/2*_ of suppression was 5 minutes longer under the continuous exposure pattern compared to the intermittent pattern, this is likely due to the timing of the onset of the light exposure relative to endogenous phase. Indeed, the continuous light exposure that started at a similar phase as the first pulse of the intermittent exposure pattern, had a suppression *t*_*1/2*_ of ~20 min, similar to the suppression *t*_*1/2*_ of ~18 min under the continuous exposure pattern. The sustained suppression of melatonin under continuous light demonstrates a sustained signal reaching the SCN and pineal. These results are in agreement with previous reports showing sustained melatonin suppression in response to polychromatic white light exposure for up to 12 h^3,48^. The sustained response is most likely driven by intrinsically photosensitive retinal ganglion cells (ipRGCs) as non-visual responses to light, including melatonin suppression, are mediated primarily by ipRGCs^4,49^. Interestingly, the rate of melatonin suppression was significantly faster under both intermittent (*t*_*1/2*_ of ~13 min) and continuous (*t*_*1/2*_ of ~18 min) light exposure as compared to the rate of decrease in melatonin levels following the cessation of melatonin synthesis estimated as *SynOff* (*t*_*1/2*_ of ~56 min)^45^. There are several possible explanations for this observation. The first possibility is that melatonin synthesis and/or secretion may not stop abruptly at *SynOff*, but rather decrease over some time, whereas synthesis and/or secretion is abruptly halted in response to bright light. This would imply that the half-life of melatonin is closer to 15 minutes than 1 hour. However, melatonin elimination half-life following intravenous administration of melatonin in humans has been estimated to be ~40 minutes. Thus, the much shorter half-life that we have observed during exposure to bright light suggests a second possibility, namely that light exposure hastens the elimination/catabolism of melatonin by an as-yet unknown mechanism. Future research will be needed to confirm the results of this exploratory analysis with appropriate measures, controls and statistical power.

Contrary to the rapid and consistent response of melatonin to continuous and intermittent light exposure, cortisol dynamics differed between continuous and intermittent light exposure. The within-participant by condition analysis revealed a significant acute increase in cortisol levels in response to continuous light exposure, followed by a slow recovery and suppression toward the end of the continuous light exposure. During intermittent bright light exposure, there was also a significant cortisol elevation as assessed by linear regression analysis of the cortisol levels during the 15-min stimuli, but no systematic changes between each light pulse. Averaging cortisol levels across individuals obscures these effects, but this might be due to the pulsatile nature of cortisol secretion^50^.

The switch from increasing to decreasing cortisol levels may be explained by the dual-drive mechanism proposed for the daily cortisol rhythm, which includes time-of-day sensitive activating and inhibitory drives^51^. Moreover, two different mechanisms have been proposed for maintaining cortisol rhythms, including a direct neural pathway originating from the SCN to the adrenal gland^24^ and molecular clocks in the adrenal cells that gate steroid synthesis and cortisol secretion^52^. Light exposure signals alter the clock gene expression patterns in the adrenal gland and are dependent on the direct sympathetic innervations of the adrenal from the SCN via the splanchic nerve^27,53,54^. Therefore, it is likely that the signaling dynamics change in either or both of these pathways with duration of exposure and time of day. An alternative explanation for this multimodal effect of light on cortisol secretion could be related to the negative feedback exerted by cortisol on its tropic hormones, CRH and ACTH. The initial activation of cortisol secretion occurring at the beginning of the light exposure could induce a subsequent decreased secretion, by altering cortisol pulsatility, in reducing either pulse amplitude or frequency. This mechanism has been described in response to a large range of cortisol-inducing stimuli, including exercise^55^, food intake^56^, ACTH1-24 administration^57^, and temperature increases^58^. A similar mechanism could be involved in response to light.

We cannot exclude that the initial increase in cortisol levels following the initiation of light exposure is secondary to a stress response, as we did not evaluate markers of stress during light exposure (e.g., psychological stress with psychometric scales, or physiological stress with ACTH or heart rate). While changes in heart rate have been reported in some studies^31,34,59^, but not in others^30^, the changes are only observed during the early morning but not at other times of the day^34^. Importantly, the change in heart rate is not associated with a change in vagal tone^59^, a reliable marker of stress response, suggesting that the change in heart rate in not secondary to a stress response. Furthermore, studies in rodent models suggest that the rise in corticosterone with light exposure is not associated with a corresponding change in ACTH^27,54^, which lends additional support to the changes in glucocorticoids observed in our study and others’ is likely not a stress response. Additional studies are required to investigate the mechanisms, however.

Aberrant light exposure is common in modern society with exposure to less light during the daytime and more at night^60^. Millions of people use their personal electronic devices before bed^61^ and millions more are engaged in shift work with frequently changing sleep-wake and light-dark cycles. While initial research suggested that very bright light was required to induce physiologic effects in humans, relatively more recent work shows that half-maximal response of 10,000 lux light is achieved with ~100 lux light including melatonin suppression, circadian phase resetting and the alerting responses^3,62^. Regular indoor intensity room light exposure prior to bed suppresses melatonin onset and shortens melatonin duration in humans^63,64^, including the light exposure from personal electronic devices^65–67^. Moreover, short duration stimuli are more effective than long duration stimuli, on a per minute basis (e.g., each minute in a single 12 min stimulus resets the pacemaker 8 times more than per minute of a 4 h stimulus^5^). Recently, millisecond flashes delivered over one hour has also been shown to induce significant phase shifts^68,69^. Our work shows that the suppressive effects of light exposure on melatonin occur rapidly whereas recovery is a relatively slower process. There are robust and complex changes in cortisol, suggesting an activation of the hypothalamic-pituitary-adrenal axis and modulation of the feedback mechanism in this pathway. Overall, the findings show that the influence of light on circadian driven endocrine physiology is dependent upon exposure patterns (continuous versus intermittent light exposure), which underscores the need to consider inter-individual differences in these responses instead of relying solely on group responses. The health consequences of the effects of intermittent light exposure, or even a single light stimulus of short duration, particularly during the biological night, are unknown, but they cannot be excluded, and deserve further investigations under highly controlled protocols.

## Data Availability

The datasets generated during and/or analyzed during the current study are available from the corresponding author on reasonable request.
